# A unique coral biomineralization pattern has resisted 40 million years of major ocean chemistry change

**DOI:** 10.1038/srep27579

**Published:** 2016-06-15

**Authors:** Jarosław Stolarski, Francesca R. Bosellini, Carden C. Wallace, Anne M. Gothmann, Maciej Mazur, Isabelle Domart-Coulon, Eldad Gutner-Hoch, Rolf D. Neuser, Oren Levy, Aldo Shemesh, Anders Meibom

**Affiliations:** 1 Institute of Paleobiology, Polish Academy of Sciences, Twarda 51/55, PL-00-818 Warsaw, Poland; 2 Dipartimento di Scienze Chimiche e Geologiche, Università di Modena e Reggio Emilia, Via Campi 103, 41125 Modena, Italy; 3 Biodiversity & Geosciences Program, Queensland Museum, South Brisbane, Qld 4101, Australia; 4 University of Washington, School of Oceanography, Box 357940, WA 98195-7940, Seattle, USA; 5 Department of Chemistry, University of Warsaw, Pasteura 1, 02-093 Warsaw, Poland; 6 MCAM UMR7245 Muséum National d’Histoire Naturelle - CNRS, Sorbonne-Universités, Paris, France; 7 The Mina & Everard Goodman Faculty of Life Science, Bar-Ilan University, 52900 Ramat-Gan, Israel; 8 Institut für Geologie, Mineralogie und Geophysik, Ruhr-Universität Bochum, D-44780 Bochum, Germany; 9 Department of Earth and Planetary Sciences, The Weizmann Institute of Science, P.O. Box 26, 76100 Rehovot, Israel; 10 Laboratory for Biological Geochemistry, School of Architecture, Civil and Environmental Engineering (ENAC), Ecole Polytechnique Fédérale de Lausanne (EPFL), CH-1015 Lausanne, Switzerland; 11 Center for Advanced Surface Analysis, Institute of Earth Sciences, Université de Lausanne, CH-1015 Lausanne, Switzerland

## Abstract

Today coral reefs are threatened by changes to seawater conditions associated with rapid anthropogenic global climate change. Yet, since the Cenozoic, these organisms have experienced major fluctuations in atmospheric CO_2_ levels (from greenhouse conditions of high pCO_2_ in the Eocene to low pCO_2_ ice-house conditions in the Oligocene-Miocene) and a dramatically changing ocean Mg/Ca ratio. Here we show that the most diverse, widespread, and abundant reef-building coral genus *Acropora* (20 morphological groups and 150 living species) has not only survived these environmental changes, but has maintained its distinct skeletal biomineralization pattern for at least 40 My: Well-preserved fossil *Acropora* skeletons from the Eocene, Oligocene, and Miocene show ultra-structures indistinguishable from those of extant representatives of the genus and their aragonitic skeleton Mg/Ca ratios trace the inferred ocean Mg/Ca ratio precisely since the Eocene. Therefore, among marine biogenic carbonate fossils, well-preserved acroporid skeletons represent material with very high potential for reconstruction of ancient ocean chemistry.

Genomic sequencing has transformed our understanding of the evolution of scleractinian corals. However, the molecular clades defined for scleractinians are difficult to reconcile with traditional taxonomic classification based on overall skeletal morphology^1^,^2^,^3^. Instead, they have been shown to be broadly consistent with recently defined micro-morphological and ultrastructural skeletal criteria^4^,^5^,^6^,^7^. In particular, distinct patterns of crystal arrangement in skeletal thickening deposits (TD, a.k.a. “fibers”) correspond well to the grouping of family-level taxa based on DNA/RNA sequencing^8^,^9^ and make it possible to taxonomically classify well-preserved fossil corals with a high level of confidence. Furthermore, a strong link between skeletal ultrastructure and molecular clade identity is consistent with a biomineralization process controlled from the gene-level with functional macromolecules (such as proteins and sulphated polysaccharides) imparting direct control over mineralogy, crystallographic properties, and certain trace-element and isotopic (e.g. δ^15^N) compositions of the resulting aragonitic structures^10^,^11^,^12^,^13^,^14^.

*Acropora* (Fig. 1a) is one of the best-studied scleractinian coral genera, with various aspects of taxonomy, biogeography, physiology, reproduction, and biomineralization investigated^15^,^16^,^17^. Complete genome sequence of *Acropora digitifera* and whole transcriptome analysis of *A*. *millepora* are available^18^,^19^. Together with *Alveopora*, *Isopora*, *Anacropora*, *Montipora*, and *Astraeopora*, *Acropora* forms a well-supported molecular clade, the family Acroporidae^3^. In the few species of *Acropora* whose fine-scale details were studied, a highly distinct, scale-like (shingle) organization of TD (Fig. 1b) has been documented^20^,^21^,^22^,^23^,^24^. However, the robustness and evolutionary stability of this skeletal feature has not been systematically examined among all *Acropora* species groups, other acroporid genera, and their fossil representatives.

We have examined the ultrastructure of 22 extant *Acropora* species representing all major morphological groups of the genus^15^ (SI Table 1). All studied specimens exhibit distinct shingle structures (consisting of overlapping, scale-like bundles of fibers) on their main skeletal surfaces, with the exception of distal portions of coenosteal spinulae and short septal spines, which are relatively smooth (e.g., Fig. 1d,e). Shingles are aligned along the extensional direction of the structures they build (e.g., Fig. 1b). Aragonite fibers within each shingle are arranged semi-parallel to the skeleton surface; tips of fibers at the growing front of shingles are extremely slender (ca. 50 nm in diameter, Fig. 1f,g). Within each shingle and in neighboring groups of shingles, fibers generally have similar orientation of the crystallographic *c*-axis (SI-Fig. 1). Shingles show incremental growth lines (3–5 μm wide) that can be observed directly on their surfaces (Fig. 1b), on polished and etched surfaces (Fig. 2c), and in thin-sections (Fig. 1c). On the skeleton surface shingles are overlapped by shingles forming just below (relative to the distal edge). Bundles of fibers that are part of individual shingles on the surface can have lengths of several hundred micrometers, strongly suggesting that their growth is continuous (Fig. 2b).

Longitudinal sections across coenosteal spinulae (Fig. 2a) show that skeleton right below the tip of the spinulae consists of two regions: dark regions (in optical transmitted light) corresponding to Rapid Accretion Deposits (RAD)^25^ (red arrows in Fig. 2a) and light regions to TD (yellow arrows in Fig. 2a), which define the width of the spinulae. The skeleton between spinulae consists of shingles (blue arrows in Fig. 2a). Because of distinct structural boundaries between spinulae and the shingles it was suggested that the shingles develop as a secondary filling deposit between already formed spinulae^24^.

We have visualized morphogenesis of *Acropora* (*A. eurystoma*) microstructural components by NanoSIMS ion microprobe and SEM imaging of skeletons pulse-labeled with ^86^Sr (Fig. 2d–f, see Methods)^26^. The first labeling pulse (12 hours during daytime) of ^86^Sr (“L1”) indicates the deposition of a continuous layer of skeleton in the distal part of the coenosteal spinulae. However, about 50–60 micrometers below the spinula tip the continuous labeling gives way to ca. 10–20 μm long, discontinuous “crescent” zones (Fig. 2f). A similar pattern is observed in subsequently labeled skeletal layers (“L2–L4”). These two characteristic features, i.e., continuous *vs.* discontinuous/crescent-like, correspond to the smooth distal part and the incipient shingles in the more proximal part of the spinulae, respectively (Fig. 1d,e). The most central part of the skeleton consists of RAD (inside dashed line in Fig. 1e) that, particularly in the lower portion of the skeleton, are visible as hollowed-out areas in polished and etched section. Growth layer L3 shows correlation between the hollowed-out areas and the relatively thickly ^86^Sr-labeled regions (regions marked with red circles in Fig. 2d,e). These observations demonstrate that skeletal tips and shingles are formed synchronously but that different dynamics of biomineralization exist in different skeletal zones. Moreover, the distinct physical shape of the shingles and a growth pattern (visualized by the ^86^Sr labeling) limited to narrow distal growth-fronts suggest compartmentalization of the biomineralization space into smaller units. This is consistent with observations of a direct 3D complementarity between the morphology of the calicoblastic cell layer and that of the skeleton at the ultrastructural level^21^,^27^, strengthening the notion of strong biological control.

The shingle ultrastructural features are pervasive throughout all *Acropora* species groups studied here (SI-Figs 2–7 and 8a–d). However, the shape and dimensions of the shingles may differ between species (Fig. 3d, SI-Table 2). In some species, such as *A*. *muricata* (type species), *A*. *echinata*, *A*. *loripes*, and *A*. *aspera* the distance between the growing fronts of overlapping shingles is relatively small (ca. 10 μm; Fig. 3d), whereas in other species (e.g., *A*. *plumosa*, *A*. *elegans* and *A*. *horrida*) these distances are well above 20 μm (e.g., Fig. 3c). The shingle units are developed in all representatives of Acroporiidae i.e., *Isopora*, *Alveopora*, *Anacropora*, *Astreopora*, and *Montipora* (Fig. 3). They are distinct and regularly developed in *Alveopora* (often are ca. 50–100 (and more) micrometers wide, Fig. 3e, also SI-Fig. 8F), *Montipora* (Fig. 3h) or less regular in *Isopora* (Fig. 3f) and *Anacropora* (Fig. 3g).

Among non-acroporid coral taxa, shingle-like structures are only exceptionally observed. The few exceptions include some agariciids^7^, a group phylogenetically closely related to Acroporidae (SI-Fig. 10a). In Flabellidae, which belongs to the same superclade Complexa as acroporids quite similar, albeit larger shingles can be observed (SI-Fig. 10b). In all other corals (Robust and Basal superclades) shingle-like structures bearing close resemblance to those of the Acroporidae are absent (SI-Fig. 10c).

Among the fossils, the oldest *Acropora* skeleton dates from Paleocene (ca. 59–56 Ma) deposits in Somalia^28^. These samples are, however, completely recrystallized and micro-scale details are not discernible. Here, we have examined for the first time the microstructures of exceptionally preserved *Acropora* fossils from several localities whose ages range from Eocene to Miocene (ca. 48 to 16 Ma, respectively; SI Table 3). Samples were initially screened for mineralogy with micro-Raman spectrometry and only skeletons with purely aragonitic thickening deposits were selected for further study (Fig. 4g,h, SI-Fig. 9). The microstructure of these fossil samples is indistinguishable from that of modern representatives of *Acropora*. For example, in regions with a well-preserved shingle texture, distal parts of skeletal protrusions (spinulae, septa) are relatively smooth (Fig. 4f). Occasionally, even details rarely preserved in fossil samples, such as rows of attachment scars of the soft tissue (imprints of desmocyte cells), can be observed with distributions indistinguishable from those in modern representatives of *Acropora* (Fig. 4c,f). In transverse thin sections of fossil coral branches, features identical to shingles of extant *Acropora* are observed: well defined bundles of fibers have lengths of several hundreds of micrometers and incremental growth lines every ca. 5 μm (Fig. 5e). In addition, a distinct concentric pattern of shingles is developed around the sections of spinulae (Fig. 5a–d). These observations indicate that the skeletal formation process was strongly biologically controlled and that skeletal micro/ultra-structures are reliable indicators of phylogenetic relations among scleractinian corals. The remarkable evolutionary stability of the *Acropora* biomineralization pattern exists despite major global geochemical fluctuations, from greenhouse (high pCO_2_) conditions and low seawater Mg/Ca (calcitic seas) in the Eocene to icehouse (low pCO_2_) conditions and rapidly increasing Mg/Ca (aragonite seas) during the Oligocene-Miocene (Fig. 6).

Mg/Ca ratios were measured in shingled thickening deposits by NanoSIMS in well-preserved fossils and extant samples of *Acropora*. Figure 6 shows measured average Mg/Ca ratios in *Acropora* skeletons plotted along with measurements previously obtained from a diverse suite of modern and fossil, non-acroporid corals^29^. The reconstructed seawater Mg/Ca ratios from fossil *Acropora* are generally consistent with the seawater Mg/Ca history derived from other fossil corals (grey circles) and other archives for the last 50 My^29^. However, the *Acropora* Mg/Ca ratios display significantly less scatter and trace a rapid increase of the inferred Mg/Ca ratio of the seawater from the Eocene (~1.5 mmol/mol) to the present (~5.2 mmol/mol), which is commonly thought to include a transition from calcite to aragonite seas^30^.

It has been suggested that scleractinians exert only partial control over their skeletal mineralogy. In laboratory experiments, *Acropora* (*A*. *cervicornis*) was reported^30^ to produce calcite in progressively higher percentages with reduction of the ambient Mg/Ca ratio below 3.5. In contrast, our results suggest that the *Acropora* lineage has maintained remarkable evolutionary stability with regard to both skeletal mineralogy and biomineralization pattern despite the major Mg/Ca fluctuations in the Cenozoic. Along with observations that extant corals are capable of up-regulating pH at the site of calcification, thereby enhancing their resilience to the effects of ocean acidification^31^, our study of fossil Acroporidae indicates that these corals can also accommodate long-term, i.e., relatively slow Mg/Ca fluctuations. Acroporids continued to form aragonitic skeletons across the Paleocene-Eocene and Oligocene-Miocene epochs but their skeletons also registered the changing Mg/Ca ratio of seawater geochemistry (Fig. 6)^32^. This suggests that a selection of samples representing the same ecological niche and phylogenetic lineage minimizes the scatter from “vital” effects. Together, the observations make the Acroporidae skeletons material of highest priority for environmental reconstruction.

## Methods

### Structural analyses

Polished sections were examined using a Nikon Eclipse 80i transmitted light microscope fitted with a DS-5Mc cooled camera head. Observations were conducted in transmitted and polarized light. Crystallographic orientation of the aragonite fibers was assessed by observations in polarized light: identical interference colors or complete light extinction of bundles of fibers in polarized light indicate similar arrangement of axes of individual crystallographic domains. One specimen of *A*. *cervicornis* was also examined with the EBSD technique following established procedures^33^. Briefly, after a final polishing with diamond compound on a lead plate the surface was manually polished using a suspension of colloidal silica (SYTON) for about 25 min. Next, after cleaning with distilled water, the sample was carbon coated (0.8 sec) and examined with a FESEM (1530 Gemini, Zeiss) equipped with a Nordlys EBSD device (HKL Technology, supplied by OXFORD instruments). The operating conditions for the SEM were a beam energy of 20 kV, an aperture of 60 microns, a working distance of 25 mm, and a tilt angle of 70°. The orientation of the mapped aragonite crystals is indicated by colors: similar directions have similar color. The crystallographic axes and faces of selected areas are plotted into the lower hemisphere of a Schmidt net.

Some sections were also examined using Phillips XL20 scanning electron microscope. For these analyses, the skeletons were gently etched for ca. 10 seconds in 0.1% formic acid, and then rinsed with Milli-Q water and air-dried. After drying, the specimens were put on stubs with double sticky tape and sputter-coated with conductive platinum film.

Mineralogy of specimens of fossil *Acropora* was analysed with a LabRAM HR Raman confocal microscope (Horiba Jobin Yvon) equipped with a LPF Iridia edge filter, a 600 or 1800 groove mm^−1^ holographic grating and a 1024 × 256 pixel Peltier-cooled Synapse CCD detector. The microscope attachment was based on an Olympus BX41 system with an MPLN100x objective and a motorized software-controlled x-y-z stage. The excitation source was the second harmonic of the diode-pumped Nd:YAG laser (Excelsior-532-100, Spectra-Physics) operating at 532.3 nm with ca. 2 mW power on the sample. Raman maps were recorded at 1 s integration time with 1 μm × 1 μm spatial resolution. Calcium carbonate polymorphs show several bands attributable to internal mode vibrations of the carbonate ion and rotational and translational lattice modes. For aragonite, the most intense peak appears at 1085 cm^−1^, which is assigned to the symmetric stretching mode of the carbonate ion. The same band for calcite is only slightly shifted towards higher energy. A characteristic doublet assigned to the in-plane bending mode of CO_3_^2−^ anion is seen at ca. 701 and 705 cm^−1^ in the spectrum of aragonite. These peaks are absent in calcite. Instead, a single band is observable at 711 cm^−1^. The most convenient signals allowing identification of the polymorph are grouped in the 100–300 cm^−1^ region. These peaks, associated with lattice vibrations, appear at 203 cm^−1^ and 153 cm^−1^ for aragonite. For calcite, the bands can be found at 281 cm^−1^ and 154 cm^−1^.

### Growth and ^86^Sr labeling experiments

Apical branch fragments of *A. eurystoma* colonies approximately 5 cm long were collected by SCUBA diving from 5–6 meters depth at the coral reef near the Inter-University Institute (IUI) in Eilat, Gulf of Aqaba, Red Sea (CITES #38406). The fragments were transferred to an outdoor, shaded running seawater table at the IUI for a month acclimation before the experiment started. *A. eurystoma* coral nubbins were labeled with ^86^Sr for 12 hours in individual glass beakers containing 500 ml of seawater enriched with 10 mg/L dissolved ^86^SrCO_3_^26^,^34^. A stream of air was gently bubbled in each beaker with a Pasteur pipet to mix the labeled seawater around the nubbin and to equalize oxygen and CO_2_ levels. Successive (L1-L4) 12 h labeling pulses took place during daytime (7:00 am to 7:00 pm) separated by 36 h intervals of growth in unlabeled seawater with normal isotopic abundances. At the end of the last labeling pulse, nubbins were snap-frozen at −80 °C to stop metabolic and biomineralization processes. For skeletal analysis, coral tissue was removed using a jet of filtered seawater (Waterpik method) and was bleached (20 minutes in 5% NaClO). Skeleton was embedded in Körapox resin and polished sections of apical corallites parallel to the vertical growing axis. The ^86^Sr/^44^Ca distribution was mapped with a NanoSIMS ion microprobe, following established procedure^26^,^35^,^36^. For orientation of the ^86^Sr labeling with the skeletal microstructure, Scanning Electron Microscopy (SEM) images of mildly etched skeleton were taken using a FEI Philips XL 20 instrument. Bleached skeletal branch tips were mounted on SEM stubs and observed platinum-coated.

### Trace element analyses

Trace element (Mg/Ca) analyses were performed with a NanoSIMS ion microprobe on polished (0.25 mm diamond suspension) and gold-coated (20 nm) skeletal surfaces embedded in Körapox^®^ epoxy following established procedures^37^,^38^. A primary beam of O^−^ (40–50 pA) produced secondary ions of ^24^Mg^+^ and ^44^Ca^+^ that were transferred to the multi-collection mass-spectrometer and detected simultaneously in electron multipliers at a mass resolving power of ~5000. At this mass-resolving power, the measured secondary ions are resolved from potential interferences. The data were obtained as spot analyses after pre-sputtering (120 seconds) with the primary ions focused to a spot-size of ~400 nm and stepped across the sample surface with a step-size of 5 micrometers (SI-Fig. 11). The measured ^24^Mg/^44^Ca ratios were calibrated against analysis of a carbonate standard of known composition (OKA-C)^39^. The chemical variations recorded in the coral skeletons are much larger than both the internal and external reproducibility of the standards, which are typically less than 3% for Mg/Ca (2 standard deviations).

## Additional Information

**How to cite this article**: Stolarski, J. *et al*. A unique coral biomineralization pattern has resisted 40 million years of major ocean chemistry change. *Sci. Rep.* **6**, 27579; doi: 10.1038/srep27579 (2016).

## Supplementary Material

Supplementary Table 1

Supplementary Table 2

Supplementary Table 3

## Figures and Tables

**Figure 1:**
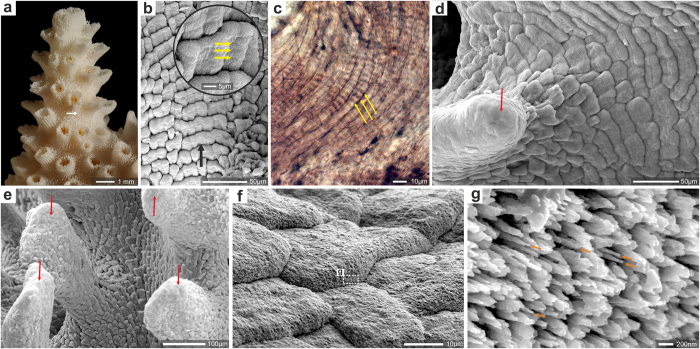
Skeleton texture and structure in Recent *Acropora*. (**a**) lateral view of a branch of *A*. *eurystoma* (ZPAL H.25/118(966)) with large axial corallite (opening at tip) and smaller lateral corallites (arrow indicates region enlarged in Fig. 1b and sectioned in Fig. 2a), (**b**) the most striking feature of the skeleton surface of *Acropora* (here *A*. *eurystoma*) is its texture in the form of regular shingles which are aligned along the extensional direction of the structures they build (black arrow). Shingles cover the entire skeleton surface except of regions of the fast growth which tend to be more smooth. The shingles show incremental growth every ca. 3–4 μm (yellow arrows in enlargement), (**c**) incremental growth lines of shingles in thin-sectioned skeleton of *Acropora muricata* (ZPAL H.25/90(535B), (**d**,**e**) distal portions of coenosteal spinulae and short septal spines (**d**, *A. cervicornis*, ZPAL H.25/12(545); (**e**) *A. eurystoma* ZPAL H.25/118(966)) are relatively smooth (red arrows) in contrast to the rest of the skeleton, which exhibits the shingle structure. (**f**,**g**) extremely slender (orange arrows in **g**) bundles of fibers form the edge of the growing front of shingles in *A. muricata*, ZPAL H.25/90(535B). Fibers are aligned parallel to the surface of the skeleton.

**Figure 2:**
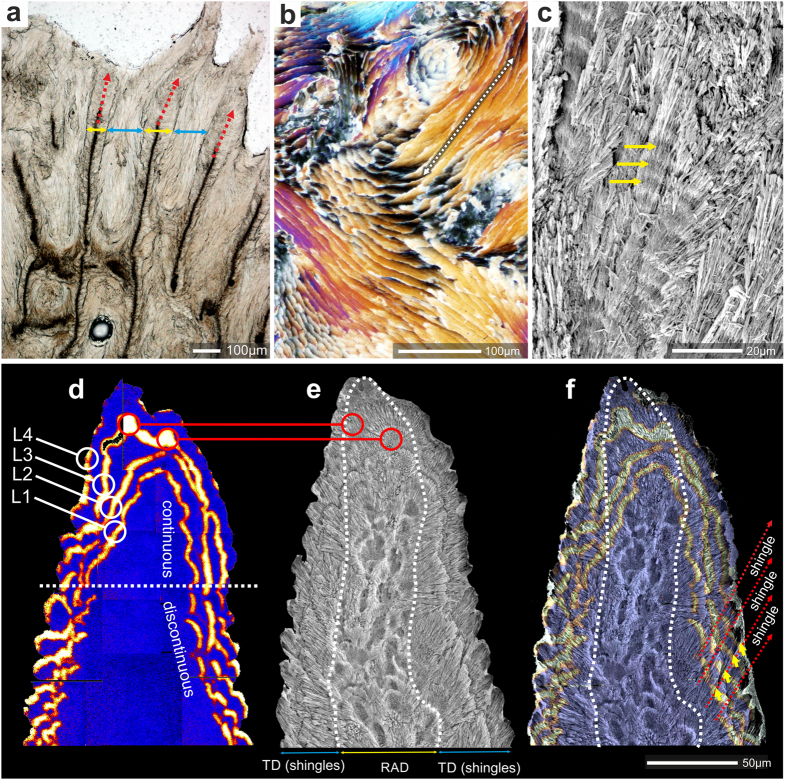
Microstructure and differentiation of shingles near the tip of coenosteal spinulae visualized by ^86^Sr labeling in Recent *Acropora* (*A. eurystoma*, ZPAL H.25/118(966)). (**a**) longitudinal section along the fast growing skeletal regions includes spinulae (yellow arrows), RAD (red arrows) and shingles (blue arrows). Ultra-thin transverse section (**b**), polarized light)) and polished, slightly etched section (**c**) reveal longitudinal sections of shingles bundles of fibers several hundreds of micrometers long (dashed arrow in (**b**)), suggesting continuous growth of individual shingles, (**d**) NanoSIMS ^86^Sr/^44^Ca isotope mosaic map, (**e**) SEM image of polished and etched sample, (**f**) NanoSIMS and SEM images overlaid. ^86^Sr-labeling pulses (12 hours, separated by 36 hours, orange-yellow color) are continuous in the distal part of the spinula and discontinuous below, where shingles are forming. Blue regions represent skeleton with normal ^86^Sr/^44^Ca ratio. Red lines with circles in d an e indicate RAD labeled with ^86^Sr. Dashed white line in e and f outlines the RAD region.

**Figure 3:**
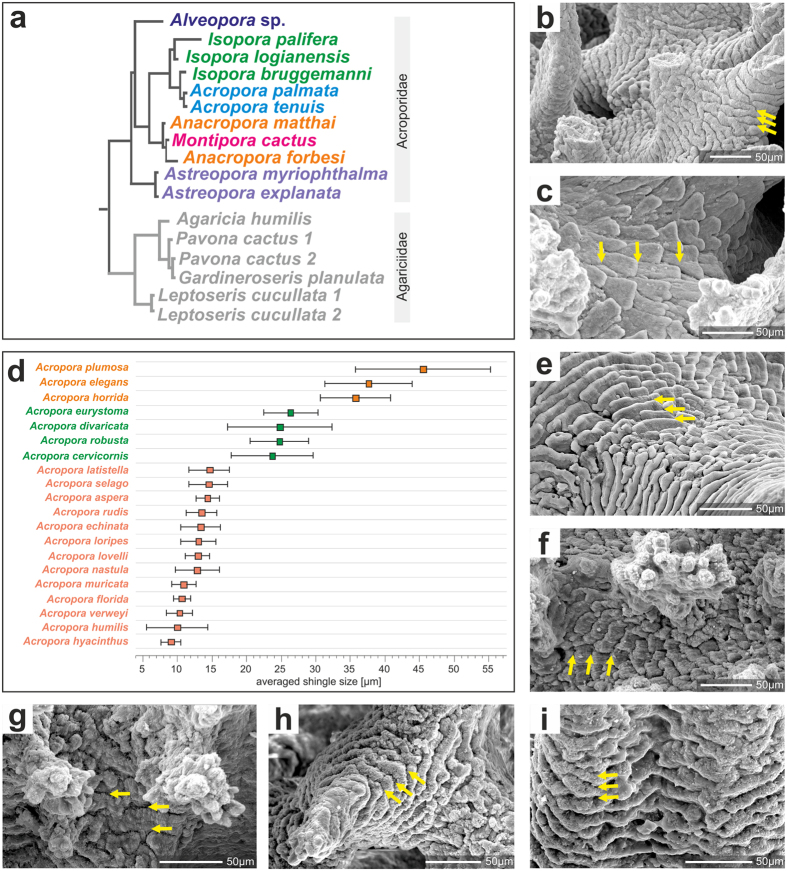
Phylogenetic relationships of Recent acroporid taxa and their skeleton surface textures. (**a**) phylogenetic tree inferred by Bayesian analysis of combined mitochondrial cox1 and cob DNA sequences^3^. All acroporids show shingled thickening deposits (**b**,**c**,**e**–**i**) but their arrangement and sizes differ among taxa (usually a species-level character), (**d**) there are 3 major size-classes of shingles within the genus *Acropora*, defined as the distance between the growing fronts of overlapping shingles (yellow arrows in **b**,**c**,**e**–**i**): (**b**), *Acropora echinata* (ZPAL H.25/83(534B)) illustrates small shingles, (**c**) *Acropora elegans* (ZPAL H.25/84(538A) illustrates large shingles. Shingles in other acroporids are illustrated with *Alveopora allingi* (**e**), ZPAL H.25/72(550A), *Isopora crateriformis* (**f**), ZPAL H.25/79(548), *Anacropora forbesi* (**g**), ZPAL H.25/87(800), *Montipora verrucosa* (**h**), ZPAL H.25/116(804), and *Astreopora myriophtalma* (**i**), ZPAL H.25/103(799). SEM images. Other examples are provided in SI-Figs 2–5.

**Figure 4:**
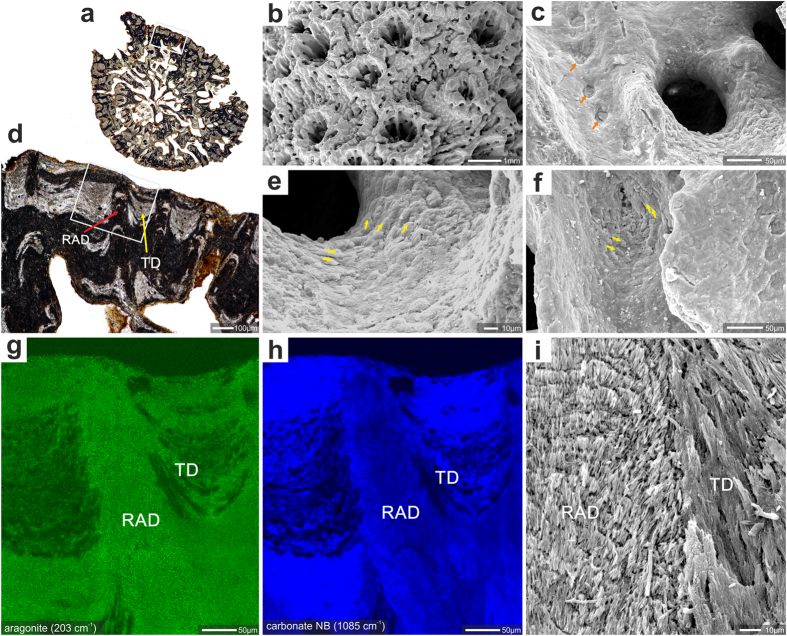
Example of good preservation of skeletal features (mineralogy, surface texture and microstructure) in fossil *Acropora*. *Acropora* sp. ZPAL H.27/20(C21), Oligocene (Chattian), Saint-Paul-lès-Dax, France. (**a**) transverse section of corallum branch with white rectangle (enlargement in (**d**)) indicating region analyzed with micro-Raman (**g**,**h**); shingled thickening deposits marked with yellow and region of rapid accretion deposits with red arrows. Surface of the branch with lateral corallites and coenosteum (**b**). Enlargment of the calice surface (**c**) with desmocyte attachment scars (orange arrows). Shingled thickening deposits, (yellow arrows in (**e**,**f**)) are still discernible, although this feature usually is the first that is eroded. Micro-Raman maps (**g**,**h**) of region indicated in d: aragonite lattice mode at 203 cm^−1^ (**g**), and carbonate vibrational mode at 1085 cm^−1^ (**h**) to show that skeleton is entirely aragonite. RAD are composed of more compact skeletal tissue in comparison to TD deposits and consequently epoxy impregnated only TD deposits (black areas), (**i**) contact zone (polished and etched surface) between RAD and TD (shingles) deposits. Note regular increment lines in RAD and elongated bundles of fibers composing TD.

**Figure 5:**
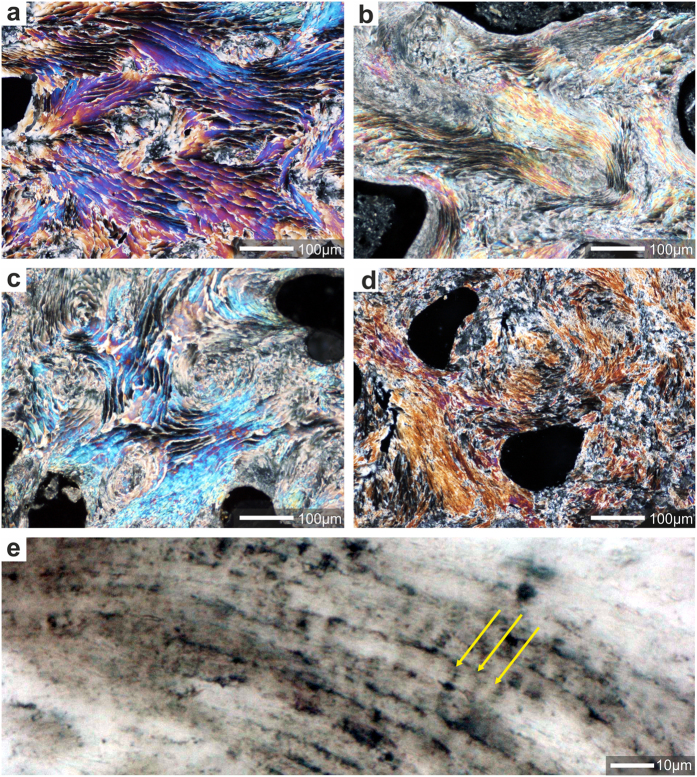
Evolutionary continuity of shingle-like biomineralization pattern in *Acropora* lineage. (**a**–**d**) Direct comparison of microstructural features of modern (**a**) and fossil (**b**–**d**) aragonite skeletons of *Acropora* showing evidence of continuous growth of shingles. Shown are ultra-thin transverse sections (polarized transmitted light, crossed Nicols) of: (**a**) extant *A*. *muricata* (ZPAL H.25/90(535B)), (**b**) Miocene (Burdigalian) *Acropora exerata* (ZPAL H.27/28(C56)), (**c**) Miocene (Aquitanian) *Acropora* sp. (ZPAL H.27/23(C54)); (**d**) Middle Eocene *Acropora alvarezi* (ZPAL H.27/18(C100)), (**e**) regular incremental growth lines (yellow arrows) in shingled thickening deposits in Miocene (Aquitanian) *Acropora* sp. (ZPAL H.27/23(C54).

**Figure 6:**
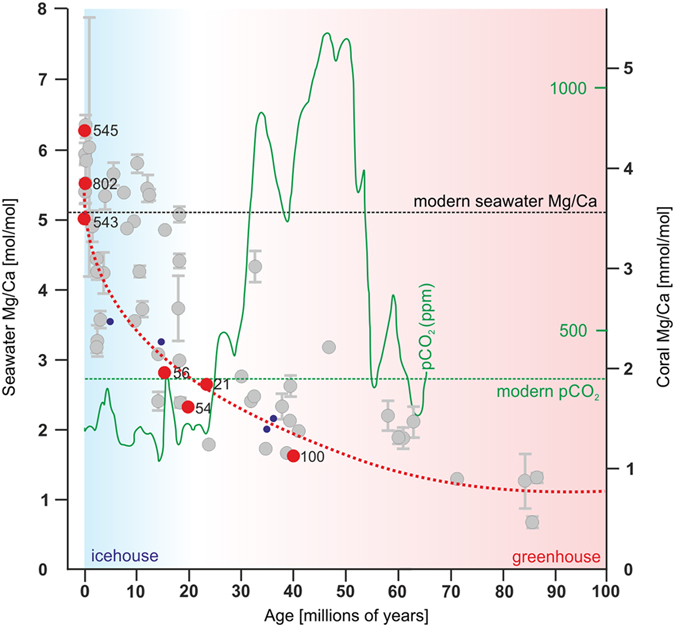
Evolutionary continuity of shingle-like biomineralization pattern in *Acropora* lineage across geochemical gradients. Seawater Mg/Ca composition during last 100 Ma inferred from diverse well preserved fossil corals (grey circles) following Gothman *et al*.^29^. Red circles represent Mg/Ca of seawater inferred from extant (ZPAL H.25/12(545), ZPAL H.25/86(802), ZPAL H.25/80(543A)) and best preserved aragonite fossil *Acropora* samples (C-SCL-56, ZPAL H.27/23(C54), ZPAL H.27/20(C21), ZPAL H.27/18(C100)). Red, dashed line is the seawater Mg/Ca reconstruction based on halite fluid inclusions (blue dots)^32^. Conventionally Mg/Ca of 2 was considered a boundary between aragonite and calcite precipitation (aragonite/calcite seas) but recent experiments^40^ show that there is a gradual and temperature-dependent shift in calcium carbonate polymorph proportions. Green curve indicates estimates of atmospheric CO_2_ reconstructed from terrestrial and marine proxies^41^. Horizontal dashed green line indicates the present-day atmospheric CO_2_ concentration (ca. 400 ppm).
